# Association between Erythrocyte Membrane Phospholipid Fatty Acids and Sleep Disturbance in Chinese Children and Adolescents

**DOI:** 10.3390/nu10030344

**Published:** 2018-03-12

**Authors:** Jun Tang, Yinkun Yan, Ju-Sheng Zheng, Jie Mi, Duo Li

**Affiliations:** 1 Department of Food Science and Nutrition, Zhejiang University, 866 Yu-hang-tang Road, Hangzhou 310058, China; scutangjun@163.com (J.T.); zhengjusheng@gmail.com (J.-S.Z.); 2 Department of Epidemiology, Capital Institute of Pediatrics, Beijing 100020, China; ykyan2011@163.com (Y.Y.); jiemi@vip.163.com (J.M.); 3 Medical Research Council Epidemiology Unit, University of Cambridge, Cambridge CB2 0QQ, UK; 4 Institute of Nutrition & Health, Qingdao University, Qingdao 266071, China

**Keywords:** erythrocytes, fatty acids, sleep disturbance, children and adolescents

## Abstract

The relationship between circulating fatty acid (FA) composition and childhood sleep disturbance remains largely unclear. We aimed to investigate the association of erythrocyte membrane FA composition with prevalence of sleep disturbance in Chinese children and adolescents. A cross-sectional survey was conducted among 2337 school-aged children and adolescents who completed a clinical assessment in Beijing, China. Presence of sleep disturbance was self-reported or parent-reported by questionnaires. Erythrocyte FAs were measured by gas chromatography, and desaturase activities were estimated by FA ratios. Multivariable-adjusted odds ratios (ORs) and 95% confidence intervals (CIs) for sleep disturbance across FA quartiles were calculated by a logistical regression model. We found higher proportions of erythrocyte phospholipid 24:0, 24:1n-9, and lower proportions of total n-3 polyunsaturated FA (PUFA), 22:5n-3 and 22:6n-3 in participants with sleep disturbance compared with those without. In the logistical regression models, significant inverse associations were found for total n-3 PUFA, 22:5n-3 and 22:6n-3, the highest versus lowest quartile ORs and 95% CIs were 0.57 (0.40, 0.82), 0.67 (0.47, 0.97) and 0.69 (0.49, 0.96), respectively. For per 1 SD difference of proportion, OR and 95% CI of prevalence of sleep disturbance was 0.91 (0.86, 0.97) for total n-3 PUFA, 0.90 (0.82, 0.98) for 22:5n-3, and 0.92 (0.86, 0.99) for 22:6n-3, respectively. No significant association was found for saturated fatty acids, monounsaturated fatty acids, n-6 polyunsaturated fatty acids or FA ratios. The present study suggested that erythrocyte n-3 PUFAs, especially 22:5n-3 and 22:6n-3, are inversely associated with prevalence of sleep disturbance in Chinese children and adolescents.

## 1. Introduction

Appropriate sleep is generally important for children’s general health and plays important roles in emotion management, physical and mental function, and immune system [1,2]. However, epidemiological studies reported that sleep disturbances, including dyssomnias, parasomnias and circadian rhythm sleep disorders, are common in children and adolescents aged 8–18 years, with reported prevalence of these sleep problems varying from 16% to 47% [3,4,5]. In previous studies, certain cases of sleep disturbances occurring in school age have been linked with declined academic performance [6,7], poor mood regulation [8,9], elevated crash rates [10,11], and even higher risk of chronic diseases such as cardiovascular disease and diabetes [12,13,14].

Fatty acids (FAs), particularly the long-chain polyunsaturated FAs (PUFAs), have been proposed to play a role in sleep processes, by exerting effects directly on neuronal membrane structure or indirectly on the dynamics of biochemical compounds (complex lipids, prostaglandins, neurotransmitters, amino acids, interleukins) necessary for the initiation and maintenance of sleep [15]. For instance, 20:4n-6 derived prostaglandin D2 (PGD2) was an inducer of sleep [16,17], while prostaglandin I2 (PGI2) was a promotor of alertness in mammalian brains [18]. The 22:6n-3 could facilitate the release of serotonin [19,20], which is known as the precursor of a sleep-inducing indolamine, melatonin. Notably, fatty acid amides including palmitamide, oleamide, and cannabinoids were also found to have sleep-inducing properties in different degrees [21,22,23].

In line with these mechanisms, clinical trials among children with attention deficit hyperactivity disorders found that supplementation of n-3 PUFAs could improve sleep quality, and reduce sleep problems [24,25]. Observational studies using biomarkers of short- (plasma and erythrocytes) or long-term (gluteal adipose tissue) dietary fat intake suggested that n-3 PUFAs were associated with lower severity of early morning awakenings [26] or obstructive sleep apnea in middle-aged adults [27], better total sleep disturbance scores in children age 7–9 years [28], and higher sleep efficiency (percentage ratio of time sleeping to time lying down) in adults aged 18–65 years [29]. Besides, gluteal adipose 18:1n-9 was also found to be positively associated with sleep efficiency in adults [30].

Although scales or parameters used for sleep quality in these studies were not uniform, it is noticeable that varied sleep outcomes were paralleled with alternated tissue FA compositions; more specifically, participants with disturbed sleep or poor sleep quality generally showed lower concentrations of blood or tissue n-3 PUFAs. Consequently, we hypothesized that a non-optimal preserve of circulating FAs is associated with occurrence of sleep disturbances. However, to date, it remains unclear whether circulating FA composition is associated with prevalence of overall sleep disturbance, especially in Children and adolescents. Thus, we performed a cross-sectional study within a large sample of school-aged children and adolescents, aimed to investigate the associations of erythrocyte saturated FAs (SFAs), monounsaturated FAs (MUFAs), PUFAs and related desaturase activities with prevalence of sleep disturbance in Chinese young population.

## 2. Method

### 2.1. Study Design and Population

We conducted a cross-sectional survey of 2837 school-aged children and adolescents who lived in Beijing, China. All the participants completed an initial clinical assessment in 2013, information on demographic, anthropometric, clinical parameters and lifestyle factors were documented into a database. After ruling out participants who have incomplete information, and who were diagnosed with cardiovascular disease, diabetes mellitus, cancers, and other endocrine diseases, 2498 participants were included for laboratory assessment on erythrocyte phospholipid FAs. Then participants with unavailable data on erythrocyte FA composition were further excluded. Finally, 2337 children and adolescents (aged 6–18 years, 52.37% boys) were included for the statistical analysis. The present study was approved by the Ethics Committee of Capital Institute of Pediatrics (Approval number: 2012062) and carried out in accordance with the Helsinki Declaration. Written consents were obtained from each participant’s parents or guardians.

### 2.2. Questionnaire

A standard questionnaire was administered by trained investigators to collect socio-demographic and lifestyle information, including age, sex, race, physical activities, sleep patterns, smoking and drinking status, dietary intake of meat, fish, dairy products, fruits and vegetables. Sleep disturbances in participants during the past month were assessed by items derived from the Chinese version of the Children’s Sleep Habits questionnaire (CSHQ) [31] and the Adolescent Health Questionnaire (AHQ) [32]. Two items were used to assess dyssomnias, including insomnia (“Do you have trouble going to sleep at night?”), sleep-disordered breathing (“Do you have any of these symptoms when sleeping: snoring, holding breath/having breathing pauses?”). Parasomnias were assessed by several typical symptoms and questions were set as “Do you have the following symptoms during sleep: moaning and limb moving, screaming, sleepwalking, bedwetting?”. Each question was provided with a yes/no choice, presence of overall sleep disturbance was adjudicated if any of the above-mentioned items were answered “yes”. Questionnaires of diet and sleep were filled out by parents for children aged 6–12 years or filled by themselves for adolescents aged 12–18 years.

### 2.3. Measurement of Anthropometric and Clinical Parameters

Participants attended the Capital Institute of Pediatrics Medical Centre in the morning following an overnight fast. Weight, height and waist circumferences were measured by trained professionals according to standard protocols. Body mass index (BMI) was calculated as weight divided by the square of height (kg/m^2^). BMI-for-age *z*-scores were calculated to define childhood adiposity in accordance with the World Health Organization Child Growth Standards [33]. Heart rate and blood pressure (BP) were measured after participants sitting still for at least 10 min, and the mean value of three measurements was adopted. Then fasting blood samples were taken and centrifuged at 2500× RCF (*g*) for 10 min at 4 °C. Plasma, buffy coat, and red blood cells were removed into separate tubes and stored at −80 °C. Serum triglycerides (TG), total cholesterol (TC), high-density lipoprotein-cholesterol (HDL-C), low-density lipoprotein-cholesterol (LDL-C) and fasting blood glucose (FBG) were determined according to standard procedures in Capital Institute of Pediatrics.

### 2.4. Laboratory Assessment for Erythrocyte Fas

Erythrocyte phospholipid FA profile of each participant was determined in Zhejiang University from January 2016 to August 2017. A volume of 0.3 mL erythrocytes was taken out and membrane phospholipids were extracted by a mixture of 10 mL chloroform/methanol (1:1 volume) (Sinopharm Chemical Reagent Co., Ltd., Shanghai, China) with eight hours of standing in −20 °C. Then 5 mL saline (Sinopharm Chemical Reagent Co., Ltd., Shanghai, China) and 5 mL chloroform (Sinopharm Chemical Reagent Co., Ltd., Shanghai, China) were added into the mixture. After shaking, the phospholipids were transferred into the chloroform layer and purified by evaporation of the solvent. Next, the purified phospholipid FAs were hydrolyzed and methylated at 70 °C for 2 h within a mixture of 3 mL methanol/vitriol (95:5) (Sinopharm Chemical Reagent Co., Ltd., Shanghai, China) and 1 mL toluene (Sinopharm Chemical Reagent Co., Ltd., Shanghai, China). At last, the methylated FAs were dissolved in hexane (Sinopharm Chemical Reagent Co., Ltd., Shanghai, China) and detected by gas chromatography equipped with flame ionization detection. FAs in samples were determined by comparing their retention times with commercial standards (Supelco Co., Ltd., Bellefonte, PA, USA) and expressed as percentage (%) of total phospholipid FAs. Desaturase enzyme activities were estimated by ratios of FAs including delta-9 desaturase (D9D-16, 16:1n-7/16:0; D9D-18, 18:1n-9/18:0), delta-6 desaturase (D6D, 20:3n-6/18:2n-6) and delta-5 desaturase (D5D, 20:4n-6/20:3n-6). 

All the laboratory staff were masked to the participants’ characteristics and samples were assessed in a random order. For quality control, the inter-assay coefficients of variation (CV) of each FAs were calculated and a value larger than 20% was regarded as reproducibility insufficient. Though over a half of the presently measured FAs had a CV >20%, means of them between monthly detected samples showed no significant difference, indicating that no batches discrepancy existed [34]. Inter-assay variation of mixture of fatty acid methyl ester standards was also monitored, the reproducibility was found to be satisfactory (all contained fatty acids had a CV <10%) (Supplementary Table S1).

### 2.5. Statistical Analysis

Continuous variables were examined for normality by Shapiro-Wilk test firstly. Continuous data with normal distribution were presented as the mean ± SD, while skewed data were presented as median and interquartile range (IQR). Basic characteristics of different study groups were compared by the *t*-test for normalized continuous variables, Mann-Whitney test for skewed variables and McNemar test for categorical variables, respectively. Further, multivariable-adjusted logistic regression models were used to investigate the associations of individual FAs and FA ratios with prevalence of sleep disturbance. FAs were divided into quartiles (Q) according to their distributions in control group. Crude odds ratios (ORs) with 95% confidence intervals (CIs) of sleep disturbance across FA quartiles were calculated firstly, then multivariable-adjusted ORs with 95% CIs were estimated in two models: model 1 was adjusted for alcohol drinking, dietary intake of meat, fish, dairy products, fruits and vegetables, since FAs in the de novo lipogenesis (DNL) pathway are affected by dietary factors [35,36]. Model 2 was adjusted for variables in model 1 plus age, sex, BMI, heart rate, systolic BP (SBP), diastolic BP (DBP), TG, TC, HDL-C, FBG, smoking status, physical exercise. Meanwhile, ORs with 95% CIs for per SD increment in individual FAs was calculated with adjustment for covariates in model 2. The consistency of overall findings was examined in subgroups defined by age, sex and body weight. Interaction test was conducted to measure whether estimated ORs significantly differed between the strata, by including simultaneously the strata factor, the FA quartiles and the interaction terms (the strata factor multiplying the FA quartiles) in the logistic regression models. All the analyses were performed by STATA version 13.0 (StataCORP, College Station LP, College Station, TX, USA). A two-tailed *p*-value < 0.05 was regarded as statistically significant. To eliminate type I error caused by multiple comparisons of 25 individual FAs and 10 ratios and sums, Bonferroni correlation was adopted and a multiple *p*-value < 0.0014 (0.05/30) was regarded as statistically significant.

## 3. Results

Basic characteristics of the participants are presented in Table 1. The mean age of participants with and without sleep disturbance was 11.7 and 10.8 years, respectively. Among the 715 participants with sleep disturbance, 31.19%, 44.90% and 53.28% were reported to have insomnia, sleep disordered breathing and parasomnias, respectively. Participants with sleep disturbance showed significantly higher values of waist circumferences, BMI, heart rate, SBP, DBP, TC and LDL-C, and tended to smoke and drink more, and eat more meat, fish and fruits than those without sleep disturbance.

The five most abundant individual erythrocyte FAs were 16:0, 18:0, 18:1n-9, 20:4n-6, 18:2n-6, which comprised nearly 80% of total fatty acids (Table 2). We found significantly higher proportions of 24:0, 24:1n-9, while significantly lower proportions of total n-3 PUFA, 22:5n-3 and 22:6n-3 in participants with sleep disturbance than those without.

ORs and 95% CIs of sleep disturbance for the highest versus lowest quartiles of FAs and desaturase activities are presented in Table 3. We found that 15:0 (OR = 0.69, 95% CI: 0.52–0.91) was inversely associated, while 24:0 (OR = 1.67, 95% CI: 1.30–2.16) and 24:1n-9 (OR = 1.48, 95% CI: 1.14–1.92) were positively associated with sleep disturbance in the crude model, but these associations were attenuated in model 1 and model 2 with adjustment for confounding factors. As for PUFAs, significant inverse associations were found for total n-3 PUFA (OR = 0.57, 95% CI: 0.40–0.82), 22:5n-3 (OR = 0.69, 95% CI: 0.48–0.99) and 22:6n-3 (OR = 0.67, 95% CI: 0.47–0.97) (model 2), and these inverse associations were consistent in the crude model and model 1.

**Figure 1:**
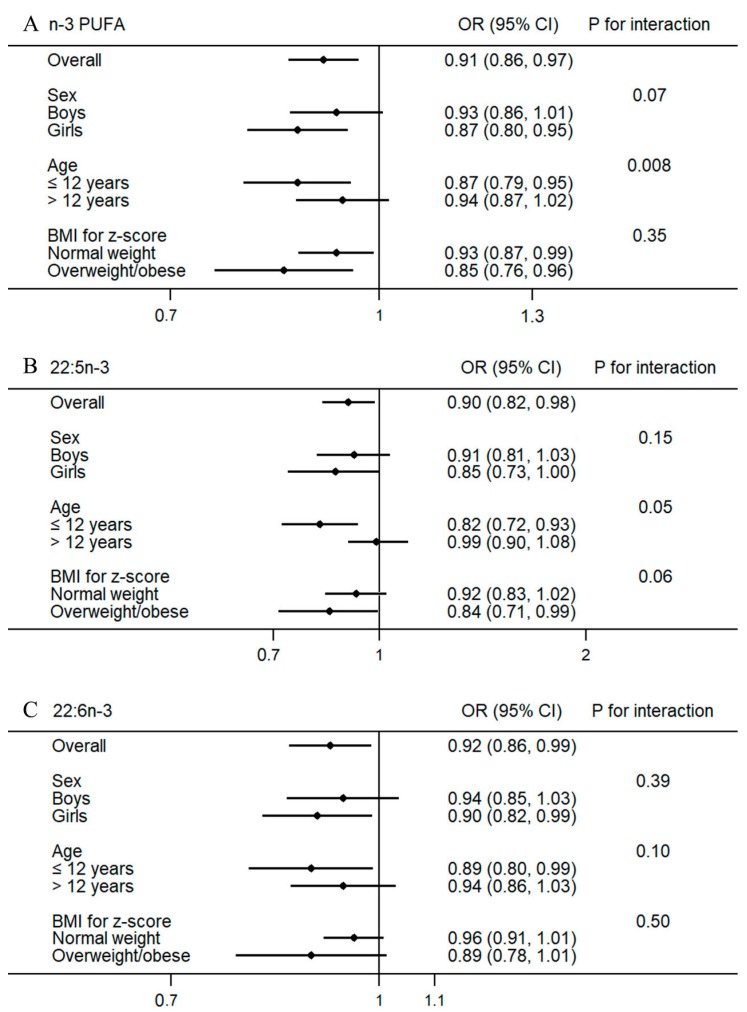
Associations of per 1 SD increment in total n-3 PUFA (**A**); 22:5n-3 (**B**) and 22:6n-3 (**C**) and with prevalence of sleep disturbance stratified by age, sex and body weight. ORs and 95% CIs were estimated with adjustment for alcohol drinking, physical activity, dietary intake of meat, fish, dairy products, fruits, vegetables, age, sex, body mass index, heart rate, systolic and diastolic blood pressure, serum triglycerides, total cholesterol, high-density lipoprotein-cholesterol, fasting blood glucose, smoking status. Abbreviation: PUFA, polyunsaturated fatty acid.

For each SD increment of total n-3 PUFA, 22:5n-3 and 22:6n-3, ORs and 95% CIs were 0.91 (0.86–0.97), 0.90 (0.82, 0.98) and 0.92 (0.86–0.99), respectively. Stratified analysis found significant interactions between age and total n-3 PUFA (*p* = 0.008), and 22:5n-3 (*p* = 0.05) (Figure 1). The inverse associations of total n-3 PUFA and 22:5n-3 were only found in participants aged 6–12 years, but not in participants aged 12–18 years. No significant interaction was found for 22:6n-3, although the overall inverse associations of 22:6n-3 with sleep disturbance turned to be marginally significant in several subgroups.

## 4. Discussion

To the best of our knowledge, the present study is the first to investigate the associations of erythrocyte FA composition and related desaturase activities with prevalence of overall sleep disturbance in Chinese children and adolescents. Children and adolescents with sleep disturbance showed higher proportions of 24:0 and 24:1n-9, but lower proportions of total n-3 PUFA, 22:5n-3 and 22:6n-3 in erythrocytes. Total n-3 PUFA, 22:5n-3 and 22:6n-3 were inversely associated with prevalence of sleep disturbance, and these findings were robust in different multivariable-adjusted models.

Previous studies have reported that different composition of FAs in diet or foods high in n-3 FAs (for example, fish) was associated with sleep quality [37,38,39]. In one cross-sectional study conducted in middle-aged Finnish men [38], higher proportions of energy-yielding nutrients (E%) of SFA and PUFA were found in participants with insomnia than in healthy participants without sleep problems, but this finding is not applicable to specific type of SFAs or MUFAs. Another intervention study investigated the effects of fatty fish consumption on several sleep parameters in males [39], after six months of intervention, sleep latency was significantly increased in control group who consumed meat, but not in fish group who consumed 300 g salmon three times per week. Although biological markers of n-3 FAs were found to be increased significantly in that study, the effect of n-3 FAs on sleep latency is not clearly understood because of the existence of other confounding factors such as vitamin D in salmon. Direct supplementation with n-3 PUFAs has also been found to have significant effects on the degree or severity of sleep disturbance. One clinical trial conducted in children with attention deficit hyperactivity disorder found that, after 12-week combined supplementation of n-3 and n-6 fatty acids as well as magnesium and zinc, percentage of participants with problems to fall asleep was reduced significantly by more than 40% [25]. However, since the trail used a mixture for treatments, it is unclear whether the beneficial effects should be attributed to n-3 PUFAs, or the other nutrients, or to them together. The stage 2 treatment in the Docosahexaenoic Acid Oxford Learning and Behavior Study found reduced Children Sleep Habits Questionnaire scores of parasomnias and total sleep disturbance in children with clinical-level sleep problems, following a 16-week treatment with 600 mg/day of 22:6n-3 compared with placebo [28]. In contrast, a 12-week multi-center randomized controlled trial among women suggested that, compared with placebo, a 1.8 g/day of omega-3 supplementation did not significantly improve any secondary measure of sleep including insomnia symptoms [40]. In observational studies, blood 22:6n-3 in adult inpatients were found to be inversely associated with severity of sleep disturbance [26] and obstructive sleep apnea [27], respectively. Blood 22:6n-3 was also found to be negatively associated with the total sleep disturbance score in 395 UK primary school students [28]. Besides, a recent US study conducted in pregnant women found that higher erythrocyte 22:6n-3 to 20:4n-6 ratio was associated with lower Pittsburgh Sleep Quality Index (PSQI), of which a score > 5 is indicative of clinically disturbed sleep [41].

Despite the various outcomes, our present findings together with the existing evidence suggest a link between n-3 PUFAs and sleep disturbance and fit well with theoretical mechanisms. On the one hand, long-chain n-3 PUFAs are major constituent in neural membranes and have been proved to play important roles in neuronal signaling pathways [42,43]. An n-3 PUFAs deficient diet (particularly 22:6n-3) could reduce concentration of serotonin in the frontal cortex of young piglets [44], and decrease the level of pineal melatonin in hamsters during the dark phase [45]. An increased concentration of 22:6n-3 in the rat hippocampus was also related to an increased content 5-hydroxytryptamine, which was a precursor of sleep-inducing agent-serotonin [46]. The n-3 PUFAs may affect the sleep-wake rhythms by regulating the metabolism of sleep relevant indolamines. However, the extent to how erythrocyte n-3 PUFAs levels reflect neuronal membrane n-3 PUFAs, and how variation of neuronal n-3 PUFAs could influence the metabolism of sleep relevant agents are not well-known and need to be further investigated. On the other hand, sleep disturbance itself can sometimes alter lipid metabolism. Recurrence of obstructive sleep apnea in patients could increase nocturnal total free fatty acids (FFAs) in plasma [47]. Similarly, sleep restriction in healthy young men resulted in an increased nocturnal and early-morning total plasma FFAs, which might be driven by two altered lipolytic hormones-growth hormone and noradrenaline [48]. In animal models, restricted sleep was found to induce lipogenesis in rat liver which was related to altered circulating lipid profiles [49,50]. However, the direct effects of disordered sleep on erythrocyte phospholipid fatty acid composition are unclear and need to be investigated by further studies.

Stratified analysis of the present study found that total n-3 PUFA and 22:5n-3 interacted significantly with age, and associations of these two FAs with sleep disturbance were discrepant in different age groups (6–12 and 12–18 years old), indicating that puberty could be one confounding factor. Pubertal maturation has been proven to be associated with later circadian phase preference [51], lower secretion of melatonin [52], and more sleep problems [53]. Except for the intrinsic factors, such as decreased secretion in sleep-related hormone, some adolescents after puberty tend to experience more detrimental environmental factors including drinking more alcohol [53], going to bed later and getting up earlier for school [54], which could eventually give rise to risk of sleep loss or disturbances [55]. Yet, how puberty and its related confounding factors might interact with erythrocyte n-3 PUFAs in sleep disturbance is unknown and should be explored by further studies. In addition, though total n-3 PUFA was found to be inversely associated with prevalence of sleep disturbance, it should be noted that this inverse association could be primarily driven by 22:5n-3 and 22:6n-3. Since the other types of individual n-3 PUFAs showed no significant association, the present finding on total n-3 PUFA should be treated with caution.

The present study has several strengths. Firstly, the sample size is larger than any of the previous studies on the same topic, and participants recruited from a school-based cross-sectional survey are more representative than those of hospital-based studies. Secondly, the present findings were robust in several models with adjustment for various covariates including lifestyle, clinical and dietary factors. Simultaneously, some limitations in our study should be noted. Firstly, FAs were assessed as percentage of total erythrocyte FAs, but not absolute concentrations. While this approach is often used in epidemiological studies and can provide a better interpretation of metabolic inter-relationships of individual FAs [56]. Secondly, since direct measurements of desaturase activities are difficult in epidemiological studies, we could only roughly estimate desaturase activities by FA ratios, which might lead to imprecise results. Thirdly, CVs were found to be relatively high for some individual FAs with a low proportion; it may be because gas chromatography is usually not sensitive enough to precisely detect low abundant FAs. Fourthly, recall bias from self-reported and parent-reported sleep disturbance could exist, and questions adopted for assessing sleep disturbance might not be robust; for example, snoring is not a great predictor for sleep apnea. Fifthly, we only focused on the association between FA profile and overall sleep disturbance, but lacked more detailed investigation on sleep apnea, insomnia and parasomnia, respectively. Sixthly, although a variety of covariates were adjusted, potential residual confounders caused by unmeasured factors could not be ruled out. Finally, because of the nature of cross-sectional studies, the present study does not demonstrate any causality.

## 5. Conclusions

The present study found that total n-3 PUFAs, 22:5n-3 and 22:6n-3 were inversely associated with prevalence of sleep disturbance in Chinese children and adolescents, and these findings were robust after adjusting for lifestyle, clinical and dietary factors. Stratified analysis showed that the inverse associations of total n-3 PUFAs and 22:5n-3 were only statistically significant in participants under 12 years of age, indicating that puberty could be one potential confounder. Further work using cohort study designs are required to determine the longitudinal associations between erythrocyte 22:5n-3 and 22:6n-3 and risk of sleep disturbance.

## Figures and Tables

**Table 1:** Demographic and clinical parameters of study participants by sleep status.

Characteristics	With Sleep Disturbance (*n* = 715)	Without Sleep Disturbance (*n* = 1622)	*p*-Value
Age, years	11.68 ± 3.31	10.79 ± 3.35	<0.001 ^a^
Sex, *n* (%)			0.45 ^b^
Boys	366 (52.16)	858 (52.90)	
Girls	349 (48.81)	764 (47.10)	
Sleep disturbance, *n* (%)			
Insomnia	223 (31.19)	-	
Sleep disordered breathing	321 (44.90)	-	
Parasomnias	381 (53.28)	-	
BMI, kg/m^2^	19.32 ± 3.68	18.59 ± 3.73	<0.001 ^a^
Waist circumferences, cm	66.12 ± 11.12	64.23 ± 10.99	<0.001 ^a^
SBP, mmol/L	109.32 ± 11.10	107.82 ± 10.67	0.002 ^a^
DBP, mmol/L	63.51 ± 7.61	62.74 ± 7.41	0.02 ^a^
Heart rate, beats/min	86.96 ± 12.00	88.79 ± 12.34	<0.001 ^a^
TG, mmol/L	0.85 (0.63–1.13)	0.81 (0.63–1.08)	0.15 ^c^
TC, mmol/L	3.89 (3.45–4.45)	4.03 (3.55–4.50)	0.002 ^c^
HDL-C, mmol/L	1.42 (1.23–1.62)	1.46 (1.29–1.67)	<0.001 ^c^
LDL-C, mmol/L	2.02 (1.69–2.46)	2.09 (1.71–2.51)	0.07 ^c^
FBG, mmol/L	5.27 (4.66–5.66)	5.29 (4.73–5.70)	0.12 ^c^
Physical exercise, min/day			0.43 ^b^
<30	468 (64.06)	1069 (65.91)	
30–60	164 (22.94)	372 (22.93)	
≥60	93 (13.01)	181 (11.16)	
Smoking status, *n* (%)			0.01 ^b^
Current	47 (6.57)	66 (4.07)	
Ever/never	668 (93.43)	1556 (95.93)	
Alcohol drinking, *n* (%)			<0.001 ^b^
Current	183 (25.59)	230 (14.18)	
Ever/never	532 (74.41)	1392 (85.82)	
Meat intake, servings/day			<0.001 ^b^
<1	246 (34.41)	728 (42.28)	
1–2	249 (34.83)	566 (32.87)	
≥2	220 (26.59)	428 (24.85)	
Fish consumption, servings/week			<0.001 ^b^
<1	321 (44.90)	565 (34.83)	
1–2	210 (29.37)	470 (28.98)	
≥2	184 (25.73)	587 (36.19)	
Dairy products, servings/day			0.37 ^b^
<1	304 (42.52)	704 (43.40)	
1–2	303 (42.38)	708 (43.65)	
≥2	108 (15.10)	210 (12.95)	
Fruit intake, servings/day			0.005 ^b^
<1	304 (42.52)	604 (37.24)	
1–2	322 (45.03)	848 (52.28)	
≥2	89 (12.45)	170 (10.48)	
Vegetables intake, servings/day			0.61 ^b^
<1	312 (43.64)	675 (41.62)	
1–2	295 (42.26)	703 (43.34)	
≥2	108 (15.10)	244 (15.04)	

Data were expressed as *n* (%), mean ± SD or median (interquartile range) where appropriate. ^a^ *p*-value from *t*-test; ^b^ *p*-value from McNemar test; ^c^ *p*-value from Mann–Whitney test. Abbreviations: BMI, body mass index; SBP, systolic blood pressure; DBP, diastolic blood pressure; TG, triglycerides; TC, total cholesterol; HDL-C, high-density lipoprotein-cholesterol; LDL-C, low-density lipoprotein-cholesterol; FBG, fasting blood glucose.

**Table 2:** Erythrocyte fatty acid composition between participant with and without sleep disturbance.

Fatty Acid (%)	With Sleep Disturbance (*n* = 715)	Without Sleep Disturbance (*n* = 1622)	*p*-Value
SFA	43.32 (40.75–49.07)	43.05 (40.41–48.41)	0.19
14:0	0.18 (0.15–0.24)	0.19 (0.15–0.24)	0.88
15:0	0.08 (0.06–0.11)	0.09 (0.07–0.12)	0.06
16:0	24.14 (22.36–29.00)	24.24 (22.49–29.09)	0.63
17:0	0.23 (0.20–0.27)	0.23 (0.20–0.27)	0.86
18:0	15.34 (14.30–16.22)	15.18 (14.20–16.07)	0.02
20:0	0.35 (0.27–0.46)	0.34 (0.27–0.47)	0.86
22:0	0.30 (0.20–0.42)	0.28 (0.19–0.41)	0.12
24:0	2.17 (1.43–3.41)	1.97 (1.31–2.81)	<0.001
MUFA	20.77 (18.85–22.88)	20.85 (18.95–22.74)	0.98
16:1n-7	0.24 (0.14–0.39)	0.22 (0.13–0.38)	0.43
18:1n-9	13.90 (12.87–15.75)	14.20 (13.07–16.09)	0.006
20:1n-9	0.29 (0.22–0.43)	0.28 (0.21–0.42)	0.21
22:1n-9	0.72 (0.52–1.02)	0.74 (0.51–1.03)	0.83
24:1n-9	4.67 (3.53–6.04)	4.23 (3.31–5.80)	<0.001
PUFA	36.37 (27.72–39.85)	36.61 (28.91–40.08)	0.20
n-3 PUFA	7.43 (6.14–8.95)	7.78 (6.49–9.38)	<0.001
18:3n-3	0.09 (0.06–0.12)	0.08 (0.06–0.12)	0.59
20:3n-3	0.11 (0.06–0.17)	0.10 (0.06–0.16)	0.25
20:5n-3	1.37 (1.03–1.79)	1.40 (1.03–1.79)	0.69
22:5n-3	1.58 (1.24–2.25)	1.68 (1.33–2.43)	<0.001
22:6n-3	4.05 (3.14–5.07)	4.29 (3.33–5.22)	<0.001
n-6 PUFA	28.73 (20.58–32.09)	29.04 (20.33–32.10)	0.55
18:2n-6	10.88 (9.27–12.17)	10.85 (9.40–12.18)	0.71
18:3n-6	0.05 (0.03–0.07)	0.05 (0.03–0.07)	0.91
20:2n-6	0.26 (0.20–0.31)	0.25 (0.19–0.31)	0.57
20:3n-6	0.96 (0.70–1.16)	0.97 (0.71–1.17)	0.58
20:4n-6	13.41 (7.90–15.51)	13.44 (7.83–15.67)	0.49
22:2n-6	0.06 (0.04–0.11)	0.06 (0.04–0.10)	0.61
22:4n-6	2.54 (1.43–3.16)	2.58 (1.47–3.13)	0.84
n-6/n-3	3.72 (2.70–4.53)	3.67 (2.53–4.55)	0.52
D9D-16 (16:1n-7/16:0)	0.0090 (0.0057–0.0144)	0.0089 (0.0057–0.0135)	0.29
D9D-18 (18:1n-9/18:0)	0.93 (0.84–1.05)	0.95 (0.86–1.08)	0.22
D6D (18:3n-6/18:2n-6)	0.0038 (0.0021–0.0066)	0.0043 (0.0024–0.0066)	0.07
D5D (20:4n-6/20:3n-6)	12.22 (10.12–14.67)	12.46 (10.20–14.86)	0.46

Data was presented as median (interquartile range). Mann-Whitney test was adopted, and a *p*-value < 0.0014 was regarded as statistically significant. Abbreviations: SFA, saturated fatty acid; MUFA, monounsaturated fatty acid; PUFA, polyunsaturated fatty acid; D9D, delta-9 desaturase; D5D, delta-5 desaturase; D6D, delta-6 desaturase.

**Table 3:** ORs and 95% CIs of sleep disturbance for highest versus lowest quartiles of FAs and FA ratios.

Fatty Acid	Crude Model	Model 1 ^a^	Model 2 ^b^
SFA	1.17 (0.91, 1.51)	1.09 (0.82, 1.44)	1.08 (0.77, 1.30)
14:0	1.06 (0.81, 1.37)	1.05 (0.80, 1.41)	1.08 (0.77, 1.52)
15:0	0.69 (0.52, 0.91) *	0.78 (0.57, 1.07)	0.83 (0.57, 1.21)
16:0	0.91 (0.71, 1.17)	0.90 (0.68, 1.20)	0.93 (0.67, 1.32)
17:0	0.98 (0.76, 1.25)	1.07 (0.81, 1.42)	1.17 (0.84, 1.63)
18:0	1.24 (0.97, 1.59)	1.30 (0.98, 1.72)	1.34 (0.96, 1.86)
20:0	0.95 (0.73, 1.23)	0.97 (0.72, 1.31)	0.95 (0.67, 1.33)
22:0	1.19 (0.91, 1.57)	1.33 (0.98, 1.82)	1.35 (0.94, 1.94)
24:0	1.67 (1.30, 2.16) *	1.43 (0.98, 2.09)	1.27 (0.90, 1.81)
MUFA	1.03 (0.81, 1.32)	1.01 (0.76, 1.33)	1.04 (0.76, 1.43)
16:1n-7	1.15 (0.90, 1.47)	1.18 (0.89, 1.57)	1.05 (0.76, 1.45)
18:1n-9	0.79 (0.62, 1.02)	0.84 (0.63, 1.11)	0.97 (0.70, 1.35)
20:1n-9	1.22 (0.95, 1.57)	1.08 (0.81, 1.47)	1.19 (0.82, 1.75)
22:1n-9	1.05 (0.81, 1.35)	0.88 (0.65, 1.18)	0.96 (0.69, 1.33)
24:1n-9	1.48 (1.14, 1.92) *	1.31 (0.95, 1.81)	1.28 (0.89, 1.83)
PUFA	0.88 (0.69, 1.13)	0.90 (0.68, 1.19)	0.91 (0.65, 1.25)
n-3 PUFA	0.61 (0.48, 0.79) *	0.61 (0.45, 0.82) *	0.57 (0.40, 0.82) *
18:3n-3	1.02 (0.79, 1.30)	0.98 (0.74, 1.30)	1.09 (0.78, 1.54)
20:3n-3	1.23 (0.95, 1.57)	1.24 (0.93, 1.64)	1.11 (0.80, 1.53)
20:5n-3	1.00 (0.78, 1.29)	0.98 (0.74, 1.30)	1.04 (0.73, 1.47)
22:5n-3	0.64 (0.49, 0.82) *	0.69 (0.51, 0.94) *	0.67 (0.47, 0.97) *
22:6n-3	0.77 (0.60, 0.98) *	0.71 (0.53, 0.95) *	0.69 (0.49, 0.96) *
n-6 PUFA	0.99 (0.77, 1.28)	1.03 (0.77, 1.37)	1.05 (0.76, 1.46)
18:2n-6	0.93 (0.73, 1.20)	0.93 (0.71, 1.23)	0.93 (0.67, 1.29)
18:3n-6	0.83 (0.66, 1.04)	0.92 (0.71, 1.19)	1.02 (0.75, 1.37)
20:2n-6	1.08 (0.84, 1.39)	0.96 (0.72, 1.29)	0.98 (0.69, 1.40)
20:3n-6	0.93 (0.72, 1.19)	0.85 (0.63, 1.13)	0.84 (0.60, 1.17)
20:4n-6	0.92 (0.71, 1.19)	1.06 (0.79, 1.42)	1.01 (0.72, 1.42)
22:2n-6	1.10 (0.86, 1.41)	1.16 (0.88, 1.53)	1.28 (0.94, 1.76)
22:4n-6	1.00 (0.78, 1.28)	1.04 (0.78, 1.38)	1.04 (0.75, 1.45)
Ratios			
n-6/n-3	1.11 (0.86, 1.44)	1.07 (0.79, 1.44)	1.13 (0.79, 1.62)
D9D-16 (16:1n-7/16:0)	1.19 (0.93, 1.53)	1.13 (0.85, 1.31)	0.99 (0.72, 1.37)
D9D-18 (18:1n-9/18:0)	0.78 (0.61, 1.01)	0.89 (0.67, 1.18)	0.95 (0.69, 1.32)
D6D (18:3n-6/18:2n-6)	0.89 (0.69, 1.15)	0.98 (0.74, 1.30)	1.13 (0.81, 1.57)
D5D (20:4n-6/20:3n-6)	0.88 (0.69, 1.14)	0.95 (0.71, 1.27)	0.99 (0.70, 1.40)

ORs and 95% CIs were estimated by logistical regression models. ^a^ Model 1 was adjusted for alcohol drinking, dietary intake of meat, fish, dairy products, fruits and vegetables; ^b^ Model 2 was adjusted for variables in model 1 plus age, sex, BMI, waist circumference, heart rate, systolic and diastolic blood pressure, serum triglycerides, total cholesterol, high-density lipoprotein-cholesterol, fasting blood glucose, smoking status, physical exercise; * Means statistically significant. Abbreviations: OR, odd ratio; CI, confidence interval; SFA, saturated fatty acid; MUFA, monounsaturated fatty acid; PUFA, polyunsaturated fatty acid; D9D, delta-9 desaturase; D5D, delta-5 desaturase; D6D, delta-6 desaturase.

## Supplementary Materials

The following are available online at http://www.mdpi.com/2072-6643/10/3/344/s1. Table S1: Percentage coefficient of variation of 25 fatty acids.
