# Supplementary material for: Association between Erythrocyte Membrane Phospholipid Fatty Acids and Sleep Disturbance in Chinese Children and Adolescents
Source: Nutrients. 2018 Mar 12;10(3):344. doi: 10.3390/nu10030344 (PMC5872762; doi:10.3390/nu10030344)
Supplement: Supplementary file 1 [file nutrients-10-00344-s001.pdf]

Table S1. Percentage coefficient of variation of 25 fatty acids.

Fatty acid	Mix Std[a]			Samples			
	Mean	SD	CV (%)	Mean	SD	CV (%)	P[b]
14:0	3.69	0.21	5.69	0.20	0.07	35.00	0.51
15:0	3.62	0.18	4.97	0.10	0.03	33.33	0.62
16:0	3.60	0.17	4.72	25.69	3.77	14.67	0.13
16:1n-7	3.59	0.17	4.73	0.27	0.08	29.6	0.64
17:0	3.53	0.17	4.82	0.24	0.05	20.83	0.07
18:0	3.37	0.17	5.04	15.07	1.72	11.41	0.09
18:1n-9	3.56	0.16	4.49	15.57	1.52	9.76	0.31
18:2n-6	3.52	0.13	3.69	10.67	1.74	16.31	0.33
18:3n-6	3.48	0.12	3.45	0.06	0.02	33.33	0.73
18:3n-3	3.43	0.12	3.50	0.10	0.03	30.00	0.11
20:0	3.54	0.16	4.52	0.39	0.09	23.07	0.14
20:1n-9	3.46	0.12	3.47	0.33	0.08	24.24	0.19
20:2n-6	3.46	0.11	3.18	0.25	0.06	24.00	0.22
20:3n-6	3.43	0.10	2.92	0.94	0.23	24.47	0.06
20:3n-3	3.50	0.14	4.00	0.11	0.03	27.27	0.30
20:4n-6	3.37	0.09	2.67	11.88	2.33	19.61	0.18
20:5n-3	3.66	0.22	6.01	1.46	0.27	18.49	0.005
22:0	3.32	0.29	8.73	0.31	0.07	22.58	0.17
22:1n-9	3.50	0.33	9.43	0.82	0.22	26.83	0.14
22:2n-6	2.79	0.26	9.32	0.08	0.03	37.5	0.25
22:4n-6	3.18	0.22	6.92	2.34	0.41	17.52	0.16
22:5n-3	3.04	0.18	5.92	1.90	0.35	18.42	0.003
22:6n-3	2.85	0.28	9.82	4.24	0.71	16.75	0.10
24:0	3.34	0.32	9.58	2.31	0.43	18.61	0.51
24:1n-9	3.54	0.24	6.78	4.72	0.65	13.77	0.006

[a] Mixture of fatty acid methyl ester standards, fresh batches of mixtures were prepared every 1 months. [b] Oneway ANOVA between monthly detected samples. Abbreviation: SD, standard deviation; CV, coefficient of variation.
